# Neurectomy as a Practical Operation*Read before the United States Veterinary Medical Association.

**Published:** 1894-11

**Authors:** S. J. J. Harger


### THE JOURNAL

OF

### COMPARATIVE MEDICINE AND VETERINARY ARCHIVES.

VOL. XV. NOVEMBER, 1894. No. 6.

### NEURECTOMY AS A PRACTICAL OPERATION.*

By S. J. J. HARGER, V. M. D.

In veterinary surgery, we find a vast field which is still unexplored. We find, among the list of major surgical operations, many which we perform, as it were, empirically, without inquiring what the actual results in a large number of cases are, or what percentage are successful. One of these is neurectomy. The term *neurotomy* is generally misapplied. It simply means a section of the nerve, whilst the word *neurectomy*, which is the correct one, means a resection of a portion of the nerve trunk, as it is ordinarily practiced. I have always been an advocate of this operation, but not unjustly so, and have always advocated it without reservation whenever the indications called for it; in fact, so much so, as to cause sometimes a smile of amusing comment from some of my professional friends whenever I did so. I do not propose to enter into many theoretical details on the subject, but rather consider it from its more practical aspect.

Great diversity of opinion exists among professional men as to the value and the results that are to be obtained from neurectomy. Some recommend it very indiscriminately, for all varieties of lesions, without first inquiring into the proper indications; this has necessarily militated against its application and brought it into disrepute. If the surgeon misapplies it, if he practices it when it is contra-indicated, not the operation but the operator should be blamed. Others, very ill-advisedly, discard it almost entirely, and

---

* Read before the United States Veterinary Medical Association.

say to the owner, "Better sell your horse." It is this diversity of opinion which has led me to write this paper.

Various nerve trunks are selected according to the end in view: the *tibial* nerve in stringhalt; the *sciatic* for lesion below the hock, when it is resected about four inches above the point of the hock and opposite to the anterior border of the tendonachilles; the *median* for lesions between the knee and the fetlock; the *plantar* and the *digital* for diseases below the fetlock. I shall only speak of the last three.

Plantar Neurectomy. Indications. One should not be too hasty in operating, for I have often seen cases in which navicular disease, existing for several weeks to several months, was diagnosed, and recovery followed the use of a foot bath, a mild liniment, or "turning out" for a few weeks. The typical indication is a chronic disease, with pain and lameness, but no mechanical obstruction. Two conditions must here be considered: 1. The accuracy of the nerve supply to the diseased area. 2. Is the lameness mechanical or is it the result of pain?

Neurectomy is practiced in ringbone, sidebone, contraction of the hoof, primary and secondary, inflammatory conditions of the laminar and the velvety tissues from traumatisms, laminitis, lesion secondary to street nails, kerathocele, quittor operation, etc. Its principal indication is the so-called chronic navicular-arthritis. Any acute inflammatory condition is always a contra-indication.

Operation. The modus operandi is well known to all of us, and hence I need not go into the didactic details of the routine surgical stages. I have used cocaine, for the last four years, instead of casting the horse. A drachm of a 4 per cent. solution in distilled water is injected subcutaneously above the fetlock over the course of the plantar nerve. In only two instances was it necessary to throw the patient. In a double neurectomy, one foot is nerved before injecting the other, to avoid the physiological effects of the drug from its absorption, which sometimes makes the horse uneasy and complicates the operation. In my experience, I have never seen the sloughing effects of cocaine, which some claim to have experienced. In this manner, the foot can be extended, placed upon a box or a stool, and held by an assistant, and the nerve removed without any laborious efforts. In fact, some horses never move, even while severing the nerve.

The only instruments necessary are a pair of curved scissors, two small dissecting forceps, a bistoury, a thin probe with a sharp but not cutting extremity, a neurotome and needle and thread, or silver wire. I never use a tourniquet or compression of any kind to arrest the hemorrhage, which is insignificant when the incision is made carefully and in the proper place; on the outside of the foot, the operation is sometimes absolutely bloodless. I do not approve of the use of Dechamp's needle, nor of securing the nerve with a thread. This prolongs the operation, and may cause the horse to struggle. I simply pass the probe under the nerve trunk after dissection, with the forceps, and then make the section. The nerve should be well isolated at the upper angle of the incision, and strong downward traction made before cutting it, in order that it does not protrude into the wound, and its extremity is not involved in the cicatrix. I always use a cutaneous suture. In this manner a nerve may be removed in a fraction of a minute. As long a portion of the nerve as possible should be removed.

The only structure that can be mistaken for the nerve, and only in low operation, and even this is not excusable, is the ligamentous cord extending from the plantar cushion to the ergot, and obliquely crossing the direction of nerve in the middle of the side of the first phalanx. Drawing the thumb nail over the nerve trunk before section or the cross-section, as well as the white color and the striation, remove all doubt. The connective tissue should be irritated and torn as little as possible.

*Prognosis.* The *prognosis* varies with the disease for which the operation is performed. In an ordinary case of navicular disease it is favorable; in those cases in which there is an intense chronic lameness of long standing, contracted tendons and ligaments, the animal is hollow-chested, and the inflammatory changes in the perforans tendon are such that the latter is weakened at its attachment to the os pedis, the prognosis is less favorable. It is *good* in ringbones when there is no anchylosis, also in sidebones in which the low operation suffices in mild cases, but the high is necessary in the aggravated forms. In laminitis the prognosis is good only in very chronic cases, when the inflammatory conditions have disappeared, and the deformity of the foot is not too marked. In general, the smaller the diseased area, the less acute and the less advanced the pathological alterations in the tissues, the more favorable is the prognosis. In acute or subacute, and in many instances of chronic, laminitis I would at any moment expect the separation of the hoof. Also is the prognosis more safe for the low operation than for the high. Again, consider the conformation of the foot. In flat-footed, thin-soled, low-heeled feet much predisposed to the effects of concussion and bruises, and in narrow and contracted hoofs and opinion must be more guarded than when the exterior form is normal. Again, the nature of the work done by the animal, the pace and the conditions of the roads have their influence.

*Median Neurectomy. Median neurectomy*, or section of the median nerve of the brachial plexus, was first practiced by Peters of Berlin, in 1885, in the treatment of inflammatory alterations of the tendons in the region of the canon and chronic lamenesses in the foot.

The modus operandi, which is slightly more complicated than in the plantar operation, simply involves the same general principles of surgery and an accurate knowledge of the anatomical relation of the parts. The nerve, situated more or less deep, accompanies the posterior radial artery and vein which must not be injured. The section is made at several points:—

1. On the inner side of the elbow at the elbow joint where this nerve passes over the internal lateral ligament; at this point the nerve is covered by the sterno-aponeuroticus muscle.

2. At the upper part of the inside of the forearm before giving off the large branch to the posterior muscles of the forearm.

3. At about the upper fourth of the forearm after giving off the above branch.

I prefer the last point of operation, because the posterior radial muscles still retain their nerve supply, which does not innervate the parts below the knee that are diseased. The incision must be made about ½ of an inch behind the internal border of the radius, and about 4 inches from its upper extremity. Unfortunately, I have not had much experience with this operation; but reasoning from the anatomical nerve distribution, and this seems to be the general consensus of opinion, it is not to be recommended from our present knowledge for troubles situated below the fetlock, such as ringbone and navicular disease. It does not completely remove the sensibility on the outside half of the foot, and hence a variable amount of lameness may remain, because the external plantar is partly composed of fibres from the ulnar.

The principal indications of this operation are lesions of region of the canon, such as chronic thickening, contraction and inflammation of the perforans, its check ligament, the perforatus, the suspensory ligament, and in those unremediable posterior splints involving the preceding structures. In the thickened and contracted tendons of the draft-horse it has been especially successful and rendered the horse entirely useful.

Some of the above cases have been followed as long as a year

Below is the table showing the results of several operators:

| Disease. | Peters. | Res. | Goldmann. | Res. | Henricks. | Res. | Baldoni. | Res. | Kull. | Res. | Pellerin. | Res. | Mousser. | Res. |
|---|---|---|---|---|---|---|---|---|---|---|---|---|---|---|
| Strain of tendons... | 1 | Cured | ...... | .......... | ...... | .......... | ...... | .......... | ...... | ...... | 5 double. | Curved. | 7 | { 6 curved. 1 incomplete. |
| Strain of tendons and lesion of pastern.. | 1 | " | ...... | .......... | ...... | .......... | 2 | { 1 cured. 1 not " | ...... | .... . | 3 | { incom- plete. | 1 | Incomplete. |
| Sidebone.......... | ...... | ...... | (Int. * side.) | Curved. | ...... | .......... | 6 | { 4 imp'd 1 not 1 cured. | ...... | ...... | .......... | .......... | ...... | .................... |
| Navicular disease.. | 2 | " | 14 | { 13 curv. 1 lame in 4 mo. | 2 | { 1 acc'd't 1 no knowl'e | 1 | Cured. | ...... | ...... | .......... | .......... | .... . | .................... |
| Chronic lameness from street nail... | ...... | ...... | 1 | Cured. | ...... | .......... | ...... | .......... | ...... | ...... | .......... | .......... | ...... | .................... |

and over, and the animal was still able to do his usual work. *Complications and Sequelae of Plantar and Digital Neurectomy.* Injury to collateral artery of the canon and the digital arteries and veins or the metatarsal veins may result in degeneration of the foot.

1. *Recurrence of the lameness* may take place in from one month to several years. In fact, the horse may not go sound at any time after the operation. Again, the lameness may continue for some weeks afterwards and then disappear. This I have seen in several cases. The recurrence or continuance may result from one or more of three causes:

a. *Errors in Diagnosis.* I have no doubt but what some very obscure lamenesses are often mistaken for navicular trouble, even in some of those cases, perhaps, which I have classified in this paper. The animal is nerved, and the operation is condemned.

b. *Recurrent Nerve Supply.* M. Arloing has demonstrated in the plantar nerves of the horse recurrent fibres which establish peripheral relations between the branches of these nerves and the nerve trunks. Some of the fibres instead of terminating in peripheral corpuscles or plates in the diseased tissues, make ascending loops and reach the main trunk, through the collateral branches. Given a section of the digital nerve, the posterior terminating branch of the plantar nerve below the fetlock, the recurrent branches coming from the diseased part return by way of the collateral branches given off above the point of section, and thus a certain amount of sensation in the parts below is retained. This distribution influences the degree of lameness which remains.

c. *Regeneration of the nerve.* The nerve tissue may regenerate itself at the central end, and the two extremities thus attain physiological union. The time required for this process varies according to, or at least is modified by, the length of the portion excised. After simple section, it follows in about a month to six weeks; after excision two months to several years, an average, as generally quoted, of one to two years. The distal end becomes atrophied. The nerve regeneration is sometimes very imperfect, which may account for some cutaneous sensibility and the continued integrity of the foot, without manifesting any lameness. On the other hand, the nerve may never regenerate itself, the intervening space being occupied by a band of cicatricial tissue. This is the ideal.

2. *Effect upon the Gait.* In navicular disease, if the perforans has not contracted adhesions and the other parts of the foot are normal, digital or low neurectomy has little or no influence upon the gait. If the lesions are chronic, the gait stilty, the animal frequently stumbles, and the tendons and ligaments have become rigid and contracted, the movements will not be as free as in the normal foot. Trotting and running races have numerous neurectomized contestants which win races in fast time. Even in saddle horses, the operation in many instances has been most successful. The high operation does sometimes interfere with the speed, although the running turf has innumerable instances of such horses that are bread-winners. In fact, one would be surprised at the number of race horses that have been nerved.

3. *Growth of the Hoof.* After section of the nerve, the horn is secreted more rapidly. The foot is apt to be high-heeled and long-toed; the wall and sole are thicker, sometimes twice the normal thickness. Shoeing should be more frequent. After the low operation the foot often approaches the normal in shape, and the heels spread.

4. Accidents from pricking street nails may be more frequent, but this is very insignificant. I have seen cases in which the quarter and portion of the sole were separated by suppuration from picking up a nail, and had to be removed; but a good recovery followed. Such was a fact in case No. 10 of my operations

5. *Degeneration of the Structures of the foot and Separation of the Hoof.* It was my intention to speak in detail of the physiological phenomena of the degeneration of the foot; but I could not obtain all the necessary data in time, and will reserve this subject for an article to be published in the near future. Brown-Sequard and Chauveau have severed the sciatic nerves of rabbits without any retrograde phenomena in the leg. The first in the tissues is a vaso-motor paralysis which is only temporary, and disappears in the course of three or four days. The pathological changes in the foot, according to my observation, seem to be of an inflammatory nature—a suppurative inflammation. The causes of this condition may be two-fold; the trophic influence of the nerves and traumatisms. The former is the predisposing cause, but at times may be the direct or exciting cause; the latter is the exciting eause. By traumatisms I do not only mean accidental injuries, but those which result from the ordinary function of the foot. Injuries to the skin of the nerved foot heal with difficulty. I have seen the foot suppurate in consequence of a blister applied to the coronet. Beyond this, I shall not discuss the question here.

*Neuroma.* These are true and false. The latter is a neurofibroma and seldom cause lameness. The treatment is excision.

### STATISTICAL CLASSIFICATION OF CASES.

### HIGH NEURECTOMY.

1. Gelding. Navic., sing. Operation 1880. 1885 sound. 5 years.
2. Mare. Navic., sing. Operation 1890. Sound. 1 year.
   Mare. Lame both feet. Second operation 1891. Sound when last seen. 6 months.
3. Gelding. Ringbone, post. Operation 1887. Permanently sound. 5 years.
4. Gelding. Ringbone, ant. Operation 1890. Died from tetanus. 1 year.
5. Gelding. Ringbone, post. Operation 1890. Permanently sound when last seen. I year.
6. Gelding. Navic., doub. Operation 1882. Tendons broke down. Sound when last seen. 2 months.
7. Gelding. Navic., doub. Operation 1883. *Neuromas*, broke down. Partially sound. 2 months.
8. Gelding. Acute laminitis. Operation, January, 1886. Both arteries cut. Bad. Foot sloughed.
9. Gelding. Ringbone, hind. Operation, November, 1888. Unsuccessful.
10. Grey Pony. Navic., sing. Operation, August, 1891. Two years ago had suppuration of quarter and sole. Still sound. 4 years.
11. Gelding. Navic., sing. Operation, April, 1891. Sound. 1 year. Foot sloughed.
12. Gelding. Navic., sing. Operation, June, 1892. Still sound. 2 years.
13. Gelding. Navic., sing. Operation, October, 1892. Lame from neuroma. Did well for 6 months. Removed. Not doing well.
14. Gelding. Navic., sing. Operation, August, 1892. Still sound. 2 years.
15. Gelding. Navic., sing. Operation, February, 1889. Still working. 5½ years.
16. Mare. Navic., very lame. Operation, February, 1889. Lost foot in 8 months.
17. Gelding. Navic. Operation, April, 1889. Did well.
18. Gelding. Navic. Operation, April, 1889. Did very well. Still working. 5½ years.
19. Mare. Navic. Operation, April, 1889. Did well for 2 years.
20. Mare. Navic. Operation, May, 1889. Not lame. Still driven. 5½ years.
21. Gelding. Navic. Operation, May, 1889. Did well until owner died. Sold as sound.
22. Mare. Navic. Operation, November, 1889. Still in use. 5 years.
23. Forseman. Gelding. Navic., double. Operation, December, 1889. Did extra well. 5 years.
24. Gelding. Navic. Operation, January, 1890. Good worker for 2 years.
25. Gelding. Navic. Operation, February, 1890. Sound. 1½ years.
26. Forseman. Gelding. Navic., double. Operation, April, 1890. Then lame. Did well.
27. Gelding. Navic., double. Operation, May, 1890. Worked in city for two years, and still on farm. 2 years.
28. Gelding. Thoroughbred. Navic. Operation, May, 1890. Won two races, and sold one year later. 1 year.
29. Gelding. Navic., double. Operation, August, 1890. Very well for two years, when sold. 2 years.
30. Gelding. Navic , double. Operation, September, 1890. Driven for two years, then sold. 2 years.
31. Gelding. Navic. Operation, February, 1891. Still at work. 3½ years.
32. Gelding. Navic. Operation, February, 1891. Sold for large price one year after. 1 year.
33. Gelding. Navic. Operation, April, 1891. Worked for one year. Killed by accident. 1 year.
34. Mare. Navic. Operation, April, 1891. Broke down in one year. 1 year. Broke down.
35. Gelding. Navic., double. Operation, May, 1891. Still working. 3 years.
36. Gelding. Navic. Operation, May, 1891. March, 1894, died. Spinal meningitis. 3 years.
37. Gelding. Navic. Operation, June, 1891. Did well, sold.
38. Gelding. Navic. Operation, July, 1891. Did well.
39. Mule. Navic. Operation, July, 1891. Still working. 3 years.
40. Gelding. Navic. Operation, May, 1889. Good. At work. 5 years.
41. Gelding Navic. Operation, May, 1889. Did well.
42. Mare. Navic. Operation, May, 1889. Did well. 4 years.
43. Mare. Navic. Operation, June, 1889. Still in use. 5 years.
44. Gelding. Navic. Operation, August, 1889. Did well for 2 years.
45. Gelding. Navic. Operation, October, 1889. Well one year, then sold. 1 year.

46. Mare. Navic. Operation, August, 1887. Serviceable. 2 years.
Second operation, March, 1888. Removed neuroma. Breaking down.
Third operation, 1889. Destroyed.

47. Gelding. Navic. Operation, August, 1887. Diagnosis. (?)

48. Ge ding. Navic., double, Operation, June, 1893. Not lame at present. 1 year.

49. Gelding. Ringbone, hind. Operation, June, 1893. No improvement.
Very lame. Second operation, August, 1893. Destroyed.

50. Gelding. Ringbone, hind. Operation, May, 1894. Not lame July 18.

51. Mare. Sidebone, ext. Operation, May, 1894.
Very lame. Second operation, Ext. nerve. Sound. Sound.

52. Gelding. Navic. Very chronic and lame. Operation, January, 1890. May 23d broke down.

53. Gelding. Navic. Operation, April, 1889. In several months foot sloughed.

54. Mare. Chronic laminitis. Operation, May, 1889. Fall of 1889 not lame.

55. Mare. Navic. Operation, September, 1891. In one month feet sloughed.

56. Mare. Navic. Operation, February, 1892. About nine months, then lame. 9 months.

57. Gelding. Navic. Operation, January, 1891. Relieved one year. July, 1892, little sore. 1 year.

58. Gelding. Ringbone. Operation, January, 1891. After six months lame. 6 months.

59. Gelding. Sidebone. Operation, February, 1891. No relief.

60. Gelding. Ringbone. Operation, February, 1891. No relief.

61. Gelding. Navic. Operation, February, 1891. September, 1892, sound 1½ years.

62. Gelding. Navic. Operation, November, 1890. At present sound. 4 years.

63. Gelding. Navic. Operation, December, 1890. Relieved about (lame). 1½ years.

64. Gelding. Ringbone. Operation, July, 1892. At present sound. 2 years.

65. Gelding. Ringbone. Operation, July, 1892 Three months, then lame. 3 months.

66. Gelding. Ringbone. Operation, September, 1892. January, 1894, slightly lame. 1½ years.

67. Mare. Navic. Operation, June, 1889. April, 1890, good. 1 year.

68. Mare Navic., double. Operation, January, 1888. January, 1890, good. 2 years.

69. Mare. Navic., three years. Operation, July, 1892. Still sound (June). 2 years.

70. Gelding. Navic. Operation 1881. 6 months.

71. Gelding Navic., recent. Operation, 1882. Lost foot in fourteen days.

72. Gelding. Navic., chronic. Operation 1882. Relieved. 2 years.

73. Gelding. Navic., acute. Operation 1890. Lost foot.

74. Mare. Navic. Operation, 1892. Not lame. 2 years. Recovered from threatened separation of foot.

75. Mare. Ringbone. Operation, 1892 Sound for one year. Destroyed for a street nail.

76. Mare. Navic., chronic. Operation, 1891. Lost foot in three months.

77. Mare. Navic., chronic. Operation, 1890. Lost foot.

78. Gelding. Navic. Operation, 1892. Artery was cut. Lost foot.

79. Gelding. Navic. Operation, February, 1891, (low). Slightly relieved.
Gelding. Navic. Operation, March, 1891, (high). Not lame. July, degeneration of tendons. Destroyed.

80. Mare. Ringbone. Operation, August, 1891. Not lame. Degeneration in foot in three months.

81. Gelding. Navic. Operation, August, 1891, (low). Little improvement.
Second operation, September, 1891, (high). No lameness. Perforans and perforatus ruptured in four months.

82. Mule. Navic. Operation, February, 1892. Degeneration of foot in thirty days.

83. Gelding. Navic. Operation, March, 1892, (low). Slight relief.
Gelding. Navic. Operation, April, 1892, (high). December, working daily, not lame. 2 years.

84. Filly. Congenitall (?) ringbone. Operation, April, 1892. Permanent, April, 1894. Exostosis very much reduced. 2½ years.

85. Mule. Ringbone. Operation, May, 1892. Permanent, working daily, exostosis smaller. 2 years.

86. Gelding. Navic. Operation, July, 1893, (low). Little improvement.
Second operation, August, 1893, (high). Permanent. 1 year.

87. Mule. Exostosis or ext. side second phalanx, Operation, September, 1893, Ext. nerve. August, 1894, sound. 1 year.

88. Gelding. Ringbone, hind, second and third phalanx. Operation, September, 1891. Sound. 3 years.
89. Gelding. Enlargement flexor tendons below fetlock. Operation, October, 1892. Sound. 2 years.

---

LOW NEURECTOMY.

1. Gelding. Navic. Operation, September, 1893. Not lame. 1 year.
2. Gelding. Navic. Operation, February, 1886. Not lame when last seen. 1½ years.
3. Gelding. Navic. Operation, January, 1891. Not satisfactory. Sold six months after.
4. Mare. Navic. Operation, January, 1888. Improved, became sore. Second operation, February, 1889. Reoperated. Sold.
5. Mare. Navic. Operation, February, 1889. Did well.
6. Mare. Navic. Operation, February, 1889. Improved and serviceable.
7. Gelding. Navic. Operation, January, 1889. Not satisfactory.
8. Gelding. Navic. Operation, July, 1889. Did well when last seen. 1 year.
9. Gelding. Navic. Operation, October, 1889. Not satisfactory.
10. Gelding. Navic. Operation, July, 1889. Passed as sound after by veterinarians. 1 year.
11. Gelding. Navic. Operation, November, 1889. No history.
12. Gelding. Navic. Operation, October, 1888. Sound in 1892. 4 years.
13. Mare. Navic. Operation, 1889. Sound at present. 5 years.
14. Gelding. Navic. Operation, April, 1890. Unsatisfactory.
15. Gelding. Navic., double. Operation, October, 1890. Destroyed for blindness. 2 years.
16. Gelding. Navic., double. Operation, October, 1892. Sold as sound in six months.
17. Mare. Navic. Operation, December, 1888. Lame afterwards.
18. Gelding. Navic. Operation, April, 1889. Driving well. 5 years.
19. Gelding. Navic. Operation, February, 1890. Still working. 4 years.
20. Gelding. Navic. Operation, 1890. Sound for one year. Nerves reunited. 1 year.
21. Gelding. Navic. Operation, April, 1893. Sound for one year. Nerves reunited. 1 year.
22. Mare. Navic. Operation, February, 1889. Eight months after lame. 8 months.
23. Mare. Navic. Operation, March, 1890. Five months after lame. 8 months.
24. Mare. Navic. Operation, April, 1890. Sound at present. Trotted in 2.40. 4 years.
25. Gelding. Navic. Operation, September, 1891. Sound. 8 years.
26. Mare. Navic. Operation, March, 1892. Still sound. 2 years.
27. Stallion. Navic. Operation, April, 1893. Several months.
28. Gelding. Navic. Operation, June, 1893. Not lame till Fall.
29. Gelding. Navic. Operation, June, 1893. In five months lame. 5 months.
30. Gelding. Navic., double. Operation, July, 1893. Pacer; speedy. Not lame at present. (Died in July.) 1 year.
31. Gelding. Navic. Operation, July, 1893. Not lame at present. 1 year.
32. Gelding. Navic. Operation, July, 1893. Not lame at present. 1 year.
33. Gelding. Navic. Operation, November, 1893. Sound at present. 1 year.
34. Gelding. Navic. Operation, November, 1893. Sound at present. 1 year.
35. Gelding. Navic., double. Operation, November, 1893. Sound at present. 1 year.
36. Gelding. Navic. Operation, January, 1891. Serviceable. 3 years.
37. Gelding. Navic. Operation, 1893. Relieved one year. Some return of lameness. 1 year.
38. Gelding. Navic. Operation, 1893. Degeneration in forty-eight hours. Opposite was sloughing from high operation.
39. Gelding. Navic., double. Operation, 1892. Apparently sound. 2 years.
40. Mare. Navic. Operation, June, 1890. Permanent foot letter form. 4 years.
41. Mare. Navic., saddle mare. Operation, June, 1890. Permanent. 4 years.
42. Mare. Navic. Operation, July, 1890. Never entirely relieved. Serviceable. 4 years.
43. Mare. Navic. Operation, July, 1890. Much improved.
44. Mare. Navic. Operation, July, 1890. Much improved.
45. Gelding. Sidebones. Operation, November, 1890. Slightly improved.
46. Mare. Navic. Operation, November, 1890. Much improved.
47. Gelding. Navic. Operation, November, 1890. Died in April, 1892. 1½ years.
48. Gelding. Navic. Operation, December, 1890. Permanently. Sold. Not lame. 3 years.

49. Gelding. Navic. Operation, January, 1891. Serviceable. 3½ years.
50. Mare. Navic. Operation, January, 1891. Slightly improved.
51. Mare. Navic. Operation, February, 1891. Lost. May. Slight lameness. 3 years.
52. Gelding. Navic. Operation, March, 1891. Slightly improved.
53. Gelding. Fract. Navic. bone. Operation, March, 1891. Much relieved. Very slightly lame.
54. Gelding. Navic. Operation, April, 1891. Permanent, January, 1894. Not lame. 3 years.
55. Gelding. Navic. Operation, April, 1891. Not lame. Sold, December, 1893. 2½ years.
56. Gelding. Navic. Operation, April, 1891. Lame after sixty days.
57. Gelding. Navic. Operation, May, 1892. Doing well. 2 years.
58. Mare. Navic. Operation, May, 1892. Doing well. 2 years.
59. Mule. Ringbone. Operation, May, 1892. Doing well. 2 years.
60. Gelding. Navic. Operation, June, 1892. Much improved, but slightly lame.
61. Gelding. Navic. Operation, June, 1892. Much improved.
62. Mare. Chronic laminitis. Operation, July, 1892. Much improved.
63. Gelding. Ringbone, low. Operation, July, 1892. Little improvement.
64. Mare. Navic. Operation, July, 1892. Sound. 2 years.
65. Gelding. Punctured sole. Operation, August, 1892. Sound. 2 years.
66. Mare. Navic. Operation, September, 1892. Serviceable. 2 years.
67. Mare. Navic. Operation, September, 1892. Little lame. 2 years.
68. Gelding. Navic. Operation, October, 1892. No relief. Destroyed.
69. Gelding. Navic. Operation, October, 1892. Slightly improved.
70. Gelding. Navic. Operation, November, 1892. Much improved.
71. Mare. Ringbone. Operation, December, 1892. Slightly lame.
72. Gelding. Fract. second phalanx. Operation, December, 1892. Working daily.
73. Mare. Navic. Operation, January, 1893. Very little lame.
74. Gelding. Navic. Operation, January, 1893. Greatly improved.
75. Mare. Navic. Operation, February, 1893. Killed.
76. Gelding. Sidebones. Operation, February, 1893. Very little improvement.
77. Mule. Low ringbone. Operation, February, 1893. Little lame. Working.
78. Gelding. Navic. Operation, March, 1893. Greatly improved.
79. Mare. Navic. Operation, March, 1893. Greatly improved.
80. Stallion. Navic. Operation, April, 1893. Greatly improved.
81. Gelding. Navic. Operation, May, 1893. Slightly relieved.
82. Mare. Navic. Operation, May, 1893. Greatly improved.
83. Mare. Navic. Operation, June, 1893. Failure. Killed.
84. Gelding. Navic. Operation, June, 1893. Sold, improved.
85. Mare. Navic. Operation, July, 1893. Greatly improved.
86. Gelding. Ringbone, low. Operation, August, 1893. Not lame. Ringbone smaller.
87. Mare. Navic. Operation, May, 1891. Not lame. 3 years.
88. Mule. Navic. Operation, June, 1891. Not lame.
89. Gelding. Navic. Operation, June, 1891. Little relieved. Became quite lame.
90. Gelding. Navic. Operation, June, 1891. Not lame. 3 years.
91. Gelding. Navic. Operation, August, 1891. Slightly lame.
92. Mare. Navic. Operation, September, 1891. Slightly improved.
93. Mare. Navic. Operation, October, 1891. Serviceable. 3 years.
94. Gelding. Navic. Operation, October, 1891. Serviceable. 3 years.
95. Mule. Sidebones. Operation, October, 1891. Relieved, December, 1894. Slightly lame. 2 years.
96. Mare. Navic. Operation, February, 1892. Permanent. Not lame. 2½ years.
97. Mare. Low Ringbone. Operation, February, 1892. August, 1894, very slightly lame. Serviceable. 2½ years.
98. Mule. Navic. Operation February, 1892. Permanent. Not lame. 2½ years.
99. Gelding. Navic. Operation, March, 1892. Unsuccessful.
100. Mare. Navic. Operation, April, 1892. Slightly lame, but serviceable. 2 years.
101. Gelding. Navic. Operation, April, 1892. Slightly lame.
102. Mare. Navic. Operation, April, 1892. Still lame.
103. Mare. Navic. Operation, April, 1892. Sound six months, when sold.
104. Gelding. Navic. Operation, March, 1893. Sound eight months. 8 months.
105. Gelding. Navic. Operation, November, 1893. Permanently sound. 1 year.

106. Gelding. Navic., double. Operation, August, 1893. Permanently sound. 2 years.
107. Gelding. Navic., double. Operation, May, 1892. Not quite sound. 2 years.
108. Mare. Navic. Operation, May, 1892. Perfectly sound. 2 years.
109. Gelding. Navic. Operation, August, 1892. Serviceable. 2 years.
110. Gelding. Navic. Operation, June, 1893. No improvement.
111. Mare. Navic. Operation, July, 1893. Sound ten months. Trotted many races. 10 months.
112. Gelding. Navic. Operation, October, 1893. Sound. 1 year.

For the collection of the statistics which form the basis of this paper, I am in a great measure indebted to some of my professional friends, among whom I take pleasure to mention Drs. Budd, Eves, Felton, Harker, Hart, Hinebauch, Michener, Muir, McKillip, Ridge, Rodgers, Schrieber, Sellers, Senseman, Chas. Williams and W. L. Williams, most of whom share my opinion on this operation. I have requested these gentlemen to select their cases good, bad, or indifferent, irrespective of the results, and have endeavored to collect them in such a manner as one would meet them in a general practice. The tables contain 231 cases, and indicate the disease, the time of operation, the result, and the termination, when such is known. All cases date prior to January 1st, 1894. Let me say that these do not represent the whole of the favorable results, because many are recent, the animals are still sound or serviceable, and may remain so for some years. Thus a case which in the table is of one year's or two years' duration and still sound, may, if followed, be found to remain so for two or three years longer. This must be taken into consideration in formulating conclusions.

Of the 231 operations, 128 are digital (low), and 103 plantar (high). In 226 of them the age of the animal was as follows:

| | | | | | |
|---|---|---|---|---|---|
| 1 | 3 years. | 33 | 9 years. | 14 | 15 years. |
| 11 | 4 " | 30 | 10 " | 5 | 16 " |
| 5 | 5 " | 5 | 11 " | 1 | 17 " |
| 16 | 6 " | 23 | 12 " | 7 | 18 " |
| 20 | 7 " | 3 | 13 " | 1 | 19 " |
| 37 | 8 " | 6 | 14 " | 3 | 20 " |

5 aged 15.

1. The author does not consider the word *sound*, in its technical sense.

Distribution of the sexes:

| | |
|---|---|
| Gelding | 164 |
| Mare | 50 |
| Stallion | 2 |
| Mule | 12 |
| Pony | 1 |
| (?) | 1 |
| | 231 |

Diseases requiring operation:

| | | |
|---|---|---|
| Navicular arthritis | One foot | 163 |
| | Both feet | 31 |
| Ringbone | | 25 |
| Sidebone | | 5 |
| Fract. navic. bone | | 1 |
| Fract. second phalanx | | 1 |
| Punct. sole | | 1 |
| Enlarg. flexor tendons | | 1 |
| Laminitis, acute | | 1 |
| Laminitis, chronic | | 2 |
| | | 231 |

Period of soundness or service, and termination of the 89 high operations. Most of them, even some of the oldest, are so at the present time.

Navicular Arthritis. 68 Cases:

| | | | |
|---|---|---|---|
| 1 | 10 years. | 4 | 1½ years. |
| 1 | 7 " | 13 | 1 " |
| 3 | 5½ " | 1 | 9 months. |
| 5 | 5 " | 1 | 8 " |
| 3 | 4 " | 2 | 6 " |
| 1 | 3½ " | 1 | 4 " |
| 3 | 3 " | 1 | 3 " |
| 14 | 2 " | 2 | 2 " |

Ringbone.

| | | |
|---|---|---|
| 1 | 5 years. | |
| 1 | 3 " | |
| 1 | 2½ " | |
| 2 | 2 " | |
| 3 | 1 " | 1 then little lame. |
| 1 | 6 months. | then little lame. |
| 1 | 3 " | then little lame. |
| 2 | No improvement. | |

Sidebone.

| | |
|---|---|
| 1 | Sound. |
| 1 | No relief. |

Enlarged Flexor Tendons, below fetlock:

| | |
|---|---|
| 1 | 2 years. |
| 7 | Did well. |
| Relieved 1 | 2 years. |

| | |
|---|---|
| Acute Laminitis | 1 suppuration. |
| Chronic Laminitis | At 4 months sound. |

Return of Lameness.

| | |
|---|---|
| 1 | 2 years. |
| 2 | 1½ " |
| 2 | 1 " |
| 1 | 9 months. |
| 1 | 6 " |
| 1 | 3 " |

These two cases, reported by Dr. Oyler of Harrisburg, are not mentioned in the table:

Neuromas.

| | |
|---|---|
| 2 | 2 years. |
| 1 | 6 months. |

Degeneration of Foot.

| Breaking Down of Tendons. | | Suppuration of Foot. | |
|---|---|---|---|
| 1 | 2 years. | 1 | 1 year. |
| 2 | 1 " | 1 | 8 months. |
| 1 | 5 months. | 2 | 3 " |
| 2 | 4 " | 1 | 2 " |
| 2 Ringbone | 2 " | 2 | 1 " |
| — | | 2 | (?) |
| 8 | | 1 | 14 days. |
| | | 1 | Immediately. |
| | | 1 | Immediately. Acute laminitis. |
| | | — | |
| | | 12 | |
| | | — | |
| Total | | 20 | |

In the majority of cases the degeneration of the foot took place within the first year, and earlier than that of the tendons.

68 Cases of Neurectomy, Plantar.

| | |
|---|---|
| 48 | Sound or serviceable from 1 to 10 years. |
| 4 | Did well. |
| 13 | Degeneration of foot. |
| | " " 1 in 9 months. |
| 2 | Return of lameness. |
| | " " 1 in 6 mos., neuroma. |
| 1 | Failure. |
| — | |
| 68 | |

Of these 68 cases, 70.6 per cent. were sound or serviceable for at least from one to ten years. The minimum is one year, but as many of them are still in this condition, the average time will be still greater than is indicated by this table.

In 20 per cent., the foot degenerated; in 3 per cent., lameness returned from reunion of the nerves or neuroma.

Low (digital) Operation.

Navicular Arthritis. 98 Cases.

| | |
|---|---|
| 2 | 5 years. |
| 5 | 4 " |
| 9 | 3 " |
| 2 | 2½ " |
| 8 | 2 " |
| 3 | 1½ " |
| 15 | 1 " |
| — | |
| 44 | |
| 1 | 10 months. |
| 1 | 8 " |
| 2 | 6 " |
| 2 | 5 " |
| 1 | 4 " |
| 2 | 3 " |
| 1 | 1 " |
| — | |
| 10 | |

Serviceable, not included in the above.

| | |
|---|---|
| 2 | No date. |
| 1 | 4 years. |
| 1 | 3½ " |
| 2 | 3 " |
| 4 | 2 " |
| 10 | |
| 15 | Much improved. |
| 6 | Slightly. |
| 12 | Unsatisfactory. |
| 1 | Degeneration, 48 hours. |
| 34 | |
| 98 | |

Return of Lameness.

| | |
|---|---|
| 1 year | 3 |
| 8 months | 1 |
| 5 " | 2 |
| 3 " | 2 |
| 4 " | 1 |
| 2 " | 1 |
| [?] | 1 |
| | 11 |

The lameness returned within the first year.

In ringbone and sidebones (see table), digital (low) section does not usually effect a cure. The patient generally is relieved or improved, but some lameness or stiffness often remains.

Of a total 88 animals thus operated on, 55 per cent. were sound or serviceable for a period, and even longer if we knew the termination given in the above table 1 to 5 years. 15 per cent. were much improved, making 70 per cent. of the cases in which, if not practical soundness of locomotion, at least a decided improvement was obtained. I know of many such cases which have been passed by veterinarians as sound. 12 per cent. were unsatisfactory, and 1 per cent. degeneration of foot.

I have operated on perhaps several hundred horses during the last seven years; but, as many were hospital, and again, as in other cases, records were imperfect, so that the animal's subsequent history is not thoroughly known to date, I know of only 30 cases of my own of which I have personal knowledge to-day. They are classified below:

### High Operation.

1. Gelding. Navic. April, 1891. Inside neuroma in two months. Then sound. 3½ years.
2. Gelding. Navic. July, 1892. Sound. Fast road horse. 2 years.
3. Gelding. Navic., double. January, 1891. Sound. 3½ years.

4. Gelding. Navic., double. October, 1893. Eight months. Then lame and fee degenerating.
5. Mare. Navic., double. January, 1891. Not relieved. Near hoof suppurated in one month.
6. Gelding. Navic., double. May, 1890. Sound for two years. Then slightly sore, but serviceable. 4 years.
7. Gelding. Ringbone, low. June, 1893. Doing well, but has lost some speed. 1 year.
8. Gelding. Navic. June, 1893. Did well for one year. Was foundered and lost foot. 1 year.
9. Gelding. Navic., double. Thoroughbred. October, 1892. Won many races for one year. Lost sight of. *Neuroma.* 1 year.
10. Mare. Navic., double. May, 1890. Serviceably sound. 4 years.
11. Thoroughbred. Stallion. Navic., double. ———— Won races for one year. Lost sight of. 1 year.
12. Mule. Navic., double. December, 1892. Sold October, 1893. Sound. 1 year.
13. Mule. Navic., double. December, 1892. Foot sloughed, October, 1893.
14. Gelding. Navic., double. ———— Foot sloughed in five weeks.

### Low Operation.

1. Mare. Navic., double. January, 1889. Sound to April, 1892. 3 years.
   Mare. Navic., double. October, 1893. Lame; nerve high. Sound, 1894. Degeneration of tendons, May, 1894. 5 years.
2. Gelding. Navic. March, 1891, double. Doing well, December, 1893. 2½ years.
3. Gelding. Navic., double. July, 1891. Relieved, 2 months, neuromas. Very lame. Destroyed, March next. Tendons contracted and adherent to navicular bone. 2 months.
4. Gelding. Navic. July, 1891. About two months, *neuromas.* Little lame. 2 months.
5. Gelding. Navic. July, 1891. Sound. Removed neuromas two years afterwards. 3 years.
6. Gelding. Navic., double. January, 1892. Doing well. 2½ years.
7. Gelding. Navic., double. May, 1893.
   Gelding. Navic., double. November, 1893. Reoperated.
   Gelding. Navic., double. June, 1894. Doing well and speedy. 2 years.
8. Gelding. Navic., double. August, 1893. Doing well. 1 year.
9. Gelding. Navic. February, 1890. Sound. Died from injury. 3 years.
10. Mare. Navic. May, 1893. Serviceable. Slightly sore. 1 year.
11. Gelding. Navic. June, 1891. Sound. 3 years.
12. Gelding. Navic. July, 1891. Sound; very speedy. 3 years.
13. Gelding. Navic. September, 1892. Sound. 2 years.
14. Gelding. Navic. March, 1893. In one month lame from neuroma. 1 month.
15. Gelding. Navic. November, 1891. Sound for 18 months. Still serviceable. 1½ years.
16. Gelding. Navic. May, 1890. Sound. Died. 3 years.

Tabulated as follows:

Plantar Operation.

2..........................................4 years.
2..........................................3½ "
1..........................................2 "
1..........................................1 "
4 [not traced].............................1 "
4..........................................Degeneration foot within one year.
—
14

In 71 per cent. the operation may be said to have been successful. Two developed neuroma No. 8 lost the foot, the result of laminitis one year after the operation; degeneration of foot in 29 per cent.

Digital Operation.

| | | |
|---|---|---|
| 6 [at present] | 3 years. | [Two died.] |
| 2 " | 2½ " | |
| 2 " | 2 " | |
| 1 " | 1½ " | |
| 2 " | 1 " | |
| 3 [neuroma] | 2 months. | |

81 per cent. of these operations were satisfactory.

From the above records, what conclusions can be drawn? Do they warrant us to recommend the operation to our client, or shall we tell him in the ordinary paraphrase, "Sell your horse"? The reply, in my opinion, should be the affirmative, always, however, keeping in mind the indications and contra-indications. My results have perhaps been a little more satisfactory than those of most others, due probably to the manner of selecting the cases. The surgeon practices very few operations of which 60 to 80 are satisfactory to him and to his client, and especially with so little expense and time required until the animal is able to resume his work, and considering that without it the animal is generally worthless. Make allowance here for incorrect diagnosis and unskillfulness in operating, and the prognosis will be still more favorable.

In choosing between the plantar and the digital operations, one must be guided entirely by existing conditions, that is, the distribution of the nerve filaments to the diseased parts. The former removes the lameness more effectively, which is not so liable to recur; but the possibility of interference with the gait and degeneration of the foot are greater. In the latter, I see absolutely no danger, excepting a more frequent recurrence of the lameness, and at times incomplete removal of the pain. Whenever the local conditions warrant it, I recommend the low operation in preference to the high

The after treatment simple. Suffice it to say that the bandages should be removed only every three or four days, and the foot placed daily into a foot-bath containing corrosive sublimate or creoline. In this way I have obtained union by first intention. The patient should be at rest for the first ten days and then have gentle exercise until the end of the third week, in order to regain the normal use of the foot and to allow the blood-vessels to resume their normal condition.

